# IMPA2 blocks cervical cancer cell apoptosis and induces paclitaxel resistance through p53-mediated AIFM2 regulation: IMPA2 blocks cervical cancer cell apoptosis

**DOI:** 10.3724/abbs.2023069

**Published:** 2023-04-28

**Authors:** Kexin Xie, Lei Liu, Min Wang, Xianping Li, Bingqi Wang, Sheng Yin, Wanxin Chen, Yingrui Lin, Xiaolin Zhu

**Affiliations:** Department of Laboratory Medicine Second Xiangya Hospital Central South University Changsha 410011 China

**Keywords:** *IMPA2*, apoptosis, *AIFM2*, *p53*, paclitaxel, cervical cancer

## Abstract

Cervical cancer continues to be a concern, and the prognosis of locally advanced cervical cancer remains poor. *IMPA2* was previously identified as a potential oncogene and regulator of tumor apoptosis. In this study, we aim to further elucidate the underlying mechanisms of *IMPA2* gene in the regulation of cervical cancer apoptosis. First, we identify *AIFM2* as an upregulated gene in *IMPA2*-silenced cervical cancer cells, and inhibition of *AIFM2* reverses *IMPA2* knockdown-induced apoptosis. Further study reveals that *AIFM2* regulates cell apoptosis in a mitochondrial-dependent manner with a redistribution of mitochondrial membrane potential and intracellular Ca ^2+^ levels. However, the analysis of the STRING database and our experimental results show that *AIFM2* has little effect on cervical cancer progression and survival. Further mechanistic study demonstrates that *IMPA2* and *AIFM2* silencing inhibits apoptosis by activating p53. Meanwhile, the knockdown of *IMPA2* enhances the chemosensitivity of cervical cancer cells by strengthening paclitaxel-induced apoptosis. Based on the above results, the IMPA2/AIFM2/p53 pathway may be a new molecular mechanism for paclitaxel treatment of cervical cancer and an effective strategy to enhance the sensitivity of cervical cancer cells to paclitaxel. Our findings display a novel function of *IMPA2* in regulating cell apoptosis and paclitaxel resistance mediated by a disturbance of *AIFM2* and *p53* expression, potentially making it a novel therapeutic target for cervical cancer treatment.

## Introduction

Cervical cancer is a common health problem that seriously threatens women’s health worldwide, with approximately 12,820 new cases and 4210 deaths per year in the US [ 1, 2] . Radiotherapy and chemotherapy are still the two main treatments for cervical cancer. However, the prognosis of locally advanced cervical cancer remains poor, and treatment still results in substantial morbidity due to chemotherapy drug resistance [ 3, 4] . Therefore, alternative therapeutic strategies are needed. Recently, the clinical potential of activators of apoptotic pathways has been widely studied in treating cervical cancer [5]. Thus, better knowledge of the molecular mechanisms modulating apoptotic pathways is urgently needed.

Myo-inositol monophosphatase 2 (IMPA2) is a critical lithium-sensitive enzyme involved in phosphoinositide (PI) signalling [6]. Most studies on IMPA2 have focused on neuropsychiatric diseases and the pharmacological action of lithium [ 7, 8] . It was reported that altered *IMPA2* gene expression was closely related to calcium homeostasis in bipolar disorder [9]. Recently, Lin *et al.* [10] found that dysregulation of *IMPA2* could promote metastatic progression of clear cell renal cell carcinoma. Our previous study demonstrated that IMPA2 could play a tumor-promoting role in cervical cancer for the first time, and proteomic analysis and flow cytometry analysis of apoptosis results showed that *IMPA2* may regulate the apoptosis process of cervical cancer [11], but the underlying mechanisms are still unknown.

Apoptosis-inducing factor mitochondria associated 2 (AIFM2/FSP1) was found to be upregulated in our previous proteomics analysis when *IMPA2* gene expression was silenced in cervical cancer cells. It was reported that AIFM2 could play a role in mitochondrial stress signaling and enhance apoptosis of human lung cancer cells [12]. Tan *et al*. [13] also proved that ATF6 could aggravate acinar cell apoptosis and injury by regulating p53/AIFM2 transcription in severe acute pancreatitis. Although evidence has shown that AIFM2 may contribute to cell apoptosis, little research has focused on its role in cervical cancer. Our previous study first showed that IMPA2 might promote cervical cancer progression [11].

In this study, we revealed that silencing of IMPA2 could aggravate apoptosis by activating the p53 signaling pathway and upregulating the expression of *AIFM2* and induce paclitaxel resistance through the IMPA2/AIFM2/P53 pathway. Our results suggest that IMPA2 is a potential therapeutic target for cervical cancer treatment.

## Materials and Methods

### Cell culture and cell transfection

The cervical cancer cell line SiHa (#BNCC337881) was purchased from the Cell Bank of BeNa Culture Collection (Beijing, China). The cervical cancer cell line HeLa (#GCC-UT0002CS) was purchased from the Cell Bank of GeneChem (Shanghai, China). *IMPA2*-silenced SiHa and HeLa cells were stably constructed as previously described [11] by transfection with an expression vector expressing *IMPA2* shRNA. The sequences of shRNAs were: shIMPA2, 5′-GCCTTACAGACGATTAACTAT-3′; and shNC, 5′-TTCTCCGAACGTGTCACGT-3′. The cells were cultured in Dulbecco’s modified Eagle’s medium (DMEM; Gibco, Grand Island, USA) supplemented with 10% fetal bovine serum (FBS; Gibco) and 1% antibiotics at 37°C in an atmosphere containing 5% CO _2_. Cells treated with the pathway inhibitors PFT-α (Sigma, St Louis, USA) and AG490 (MedChem Express, New Jersey, USA) were incubated for 24 h and 48 h, respectively.

For *AIFM2* knockdown, *AIFM2* siRNAs were chemically synthesized by BioeGene (Shanghai, China), and the sequences were as follows: si-1, 5′-CCAAAUCAGUGGCUUCUAUTT-3′; si-2, 5′-GCACCGGCAUCAAGAUCAATT-3′; si-3, 5′-GCUGCCUCUCAAUGAGUAUTT-3′; and siNC, 5′-UUCUCCGAACGUGUCACG-3′. According to the manufacturer’s instructions, the cells were transfected with siRNAs using LipoHigh (Sangon Biotech, Shanghai, China). After 6 h of culture, the medium was replaced by fresh medium, and the cells were cultured for 24 h for RNA extraction and 48 h for protein extraction and other experiments.

### Tissue samples

Tissue samples for Bak and AIFM2 immunohistochemistry (IHC) staining were xenografts from mice that were injected with *IMPA2*-silenced or control SiHa cells derived from our previous study [11]. Tissue samples for *AIFM2* mRNA detection and IHC staining were obtained from three patients with written consents who underwent radical hysterectomy at the Second Xiangya Hospital, Central South University. This study was approved by the Joint Ethics Committee of the Central South University Health Authority (No. 2020046, Date: 2020/04/01) and performed in accordance with the Declaration of Helsinki.

### Flow cytometric analysis

After transfection for 6 h, the cells were incubated for 48 h at 37°C, in a 5% CO _2_ incubator. The collected cells were then rinsed twice with PBS and prepared for subsequent testing. After treating *IMPA2*-silenced SiHa and HeLa cells with 0 μM and 20 μM PFT-α (Sigma) for 24 h, apoptosis was detected using an Annexin-V-APC/PI staining kit (Biolegend, Shanghai, China) according to the manufacturer’s instructions and finally analysed on a Guava easyCyte HT flow cytometer (Millipore, Billerica, USA).

### Immunohistochemistry (IHC) analysis

The immunohistochemical staining procedure was performed using standard protocols. The following formula was used to calculate the staining positivity: IRS=intensity score×quantity score. The percentage of positive cells was divided into five score ranks: <10% (0), 10%–25% (1), 25%–50% (2), 50%–75% (3), and >75% (4). The intensity of staining was divided into four score ranks: no staining (0), light brown (1), brown (2), and dark brown (3). All the specimens were evaluated by two pathologists independently in a blinded manner. The antibodies used were as follows: anti-Bak (1:100; CST, Boston, USA) and anti-AIFM2 (1:500; Affinity Biosciences, Melbourne, Australia).

### Immunofluorescence microscopy

Immunofluorescence staining was performed using antibodies against Caspase-3 (1:500; Abcam, Cambridge, UK) and AIFM2 (1:100; Affinity Biosciences) following the previously mentioned protocol. After incubation with HRP-conjugated goat anti-rabbit IgG secondary antibody (1:1000; Abcam), the nuclei were stained with DAPI (Beyotime Biotechnology, Shanghai, China). The immunofluorescence signals were examined using an A1 fluorescence microscope (Zeiss, Oberkochen, Germany).

### Measurement of the intracellular Ca ^2+^ concentration

The mitochondrial Ca ^2+^ concentration was detected using a Rhod-2 ^AM^ probe (Maokang, Shanghai, China). Detection was carried out according to the manufacturer’s instructions, immunofluorescence signals were examined using the A1 fluorescence microscope, and ImageJ was used for fluorescence quantification.

### Measurement of mitochondrial membrane potential

The extent of mitochondrial membrane potential (MMP) loss was measured using the potentiometric cation JC-1 (MedChemExpress). Transfected HeLa or SiHa cells were incubated with JC-1 staining solution for 20 min at 37°C and examined under the A1 fluorescence microscope.

### CCK-8 cell viability assay

Cells transfected with siAIFM2 or siNC were seeded into a 96-well plate at 2×10 ^3^ cells per well with 100 μL of culture medium and cultured for 24, 48, 72, and 96 h at 37°C and 5% CO _2_. Cell viability was determined using a CCK8 assay kit (KeyGen Biotech, Nanjing, China) according to the manufacturer’s instructions. Each process was repeated three times.

### RNA isolation and quantitative real-time PCR

Total RNA was extracted using TRIzol reagent (Sangon Biotech). RNA (1 μg) was reverse transcribed into cDNA using the Transcriptor First Strand cDNA Synthesis Kit (Roche Diagnostics, Mannheim, Germany) according to the supplier’s instructions. Quantitative real-time PCR analysis was performed with a Stratagene Mx3000P qPCR system (Agilent Technologies, Santa Clara, USA) using Thunderbird qPCR Mix (Toyobo, Osaka, Japan). cDNA samples were tested in triplicate, and glyceraldehyde-3-phosphate dehydrogenase ( *GAPDH*) was used as a reference gene. The expression of genes was quantified by measuring Ct values and normalized using the 2 ^‒ΔΔCt^ method relative to *GAPDH*. The primer pairs used for qRT-PCR were designed using the Primer3 program. The primers used are shown in Table 1.

**Table:** **Table 1** Sequences of primers used in this study for qRT-PCR

Gene	Forward primer (5′→3′)	Reverse primer (5′→3′)
*AIFM2*	CAAGATCAACAGCTCCGCCTACC	CGTCGGCACAGTCACCAATGG
*BLOC1S2*	ACTGGCGACCCGGAGTGATG	GTCAGCTTCAGCAGGCTCCTTTG
*MYDGF*	CGGCGTCGTGCATTCCTTCTC	CCATTGCTCATTGGTCCCTCCTTG
*MLH1*	TCTCAGGTTATCGGAGCCAGCAC	ATCTTCCTCTGTCCAGCCACTCTC
*HERPUD1*	ATCAGGGGCTTTTGTTCCACCAC	ACAACCACTTGAGGAGCAGCATTC
*CAV1*	GCAGAACCAGAAGGGACACACAG	CCAAAGAGGGCAGACAGCAAGC
*HSF*	CGACAGTGGCTCAGCACATTCC	ACAGCATCAGGGGCGTAGAGG
*MTM-1*	ATCCAGTTGCCAGTATGCGTCAC	TCGGCTGTTGTTGCTTGATCCTG

### Western blot analysis

The cell extracts were prepared using RIPA buffer (KeyGen Biotech) containing protease inhibitors (KeyGen Biotech). Equal amounts of protein samples were subjected to 12% SDS-PAGE and transferred to PVDF membranes (Immobilon-P; Millipore, Billerica, USA). The membranes were then blotted with primary antibodies overnight at 4°C, followed by incubation with the HRP-conjugated goat anti-rabbit IgG secondary antibody (1:5000). The antibodies used were as follows: anti-AIFM2 (1:1000; Biorbyt, Cambridge, UK), anti-Bcl2 (1:2000; Abcam), anti-Bax (1:5000; Abcam); anti-Caspase3 (1:5000; Abcam), anti-Bak (1:1000; CST), anti-Cytc (1:1000; Abcam), anti-p53 (1:2000; Abcam), anti-p-mTOR (1:1000; CST), mTOR (1:1000; CST), anti-p-PI3K (1:1000; Affinity Biosciences), anti-PI3K (1:1000; Affinity Biosciences), anti-p-AKT (1:1000; CST), anti-AKT (1:1000; CST), anti-p-JAK2 (1:1000; Abcam), anti-JAK2 (1:1000; Abcam), anti-p-STAT3 (1:5000; Abcam), anti-STAT3 (1:5000; Abcam) and anti-GAPDH (1:5000; BBI, Shanghai, China). The protein bands were then visualized using enhanced chemiluminescence reagent (Bio-Rad, Hercules, USA). Band quantification was conducted using ImageJ (National Institutes of Health, Bethesda, USA).

### Statistical analysis

Data are shown as the mean±standard deviation (SD) based on at least three independent experiments. The results were analysed using SPSS 22.0 (SPSS, Chicago, USA) and GraphPad Prism 6 software (GraphPad, San Diego, USA). Two-tailed Student’s *t*-test was used to evaluate the difference between two data groups. A *P* value<0.05 was considered statistically significant.

## Results

### *IMPA2* knockdown upregulates *AIFM2* gene expression to induce apoptosis of HeLa and SiHa cells

**Figure 1:**
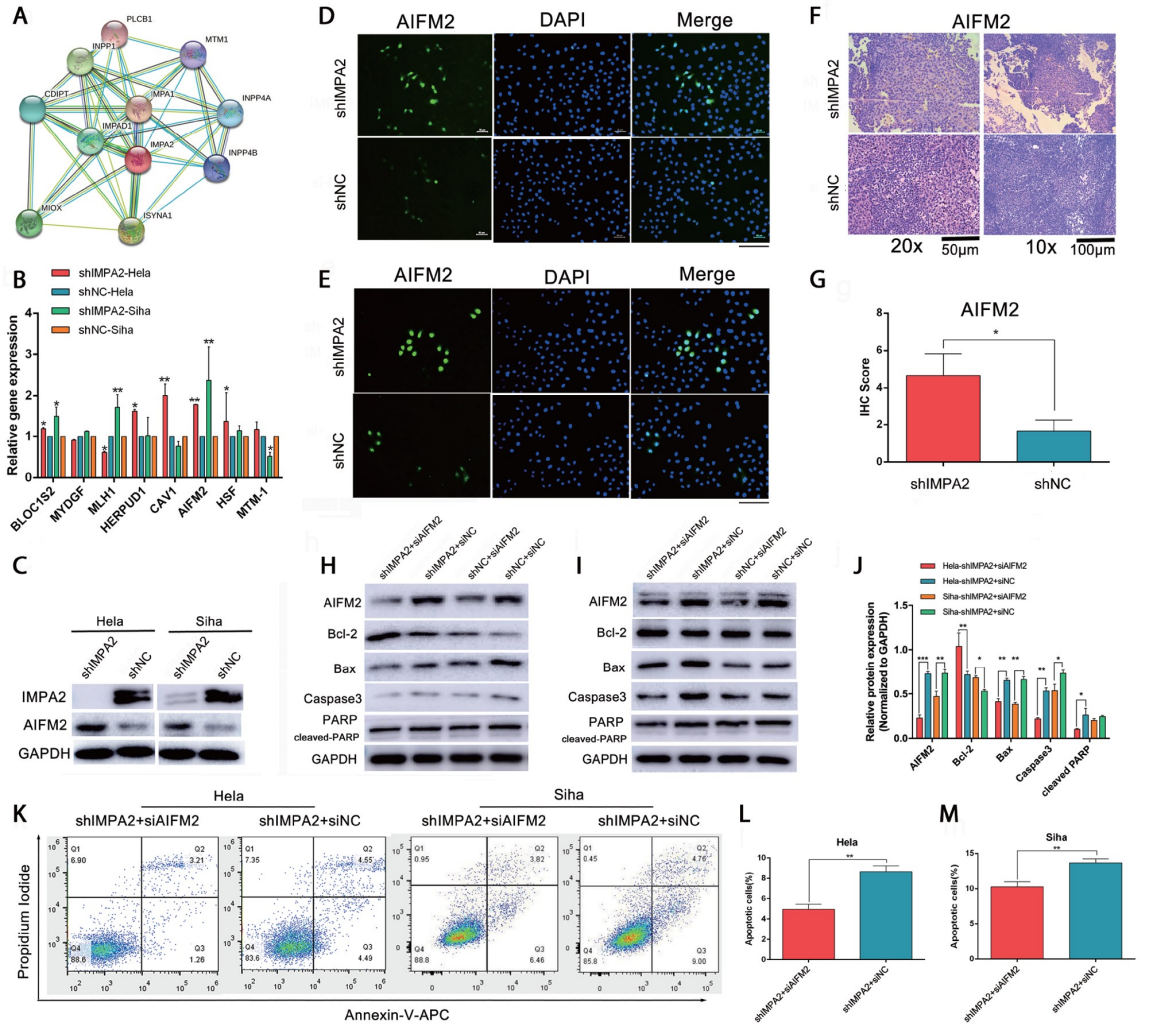
Figure 1 *IMPA2* knockdown triggers *AIFM2* expression to induce cervical cancer apoptosis To explore the molecular IMPA2-regulated process of cell apoptosis, we searched a related database and screened apoptosis-related differentially expressed proteins from proteomics. (A) The result of the STRING database is shown. (B) Eight proteins were chosen, and mRNA expression levels were measured by qRT-PCR in the shIMPA2 group and shNC group. Among them, AIFM2 was upregulated in both HeLa and SiHa cells. (C) Protein expression levels of AIFM2 were measured and quantified in the shIMPA2 group and shNC group. (D,E) The fluorescence intensity of AIFM2 was detected and observed by fluorescence microscopy. Scale bar: 50 μm. (F,G) AIFM2 expression in xenografts was detected by immunohistochemical staining, and the immunoreactivity scores of AIFM2 are shown in G. Scale bar: 100 or 50 μm. (H–J) To verify whether IMPA2 knockdown-induced apoptosis could be reversed by AIFM2 knockdown, shIMPA2, siAIFM2, shNC, and/or siNC were cotransfected into HeLa and SiHa cells. Then, apoptosis-related proteins were detected by western blot analysis and quantified using ImageJ. (K) Flow cytometry analysis of apoptosis was performed to further confirm that IMPA2 silencing-induced cell apoptosis could be restored by AIFM2 inhibition. The percentages of apoptotic HeLa (L) and SiHa (M) cells were counted, and significant differences were analysed. Data are presented as the mean±SD of three replicates. * P<0.05, ** P<0.01, *** P<0.001.

To investigate the underlying mechanisms of the modulation of *IMPA2* in the apoptotic process of cervical cancer cells, we screened 8 molecules related to apoptosis from the previous proteomic results. We created the STRING database ( http://string-db.org) to find the interacting molecules ( Figure 1A). After silencing *IMPA2*, only *AIFM2* mRNA expression was upregulated in HeLa and SiHa cells ( Figure 1B). Similar results were obtained in the protein expression level detected by western blot analysis ( Figure 1C). In addition, immunofluorescence results also showed that *IMPA2* knockdown increased *AIFM2* expression in both HeLa and SiHa cells ( Figure 1D,E). Meanwhile, IHC staining also demonstrated higher *AIFM2* protein expression in xenografts from mice after injecting shIMPA2-Siha cells ( *P*<0.05; Figure 1F,G). These results indicated that inhibition of *IMPA2* expression could upregulate *AIFM2* gene expression. Furthermore, *IMPA2* expression knockdown-induced changes in apoptosis-related proteins were reversed by cotransfecting with *AIFM2* siRNA, with an increase in Bcl-2 protein level and decreases in Bax and Caspase3 protein levels ( Figure 1H‒J). Similarly, apoptotic cells were also decreased when *AIFM2* and *IMPA2* genes were simultaneously knocked down in cervical cancer cells ( Figure 1K‒M). All these data suggested that *IMPA2* inhibition could promote apoptosis of HeLa and SiHa cells by activating *AIFM2* expression.

### Downregulation of *AIFM2* represses mitochondria-dependent apoptosis

**Figure 2:**
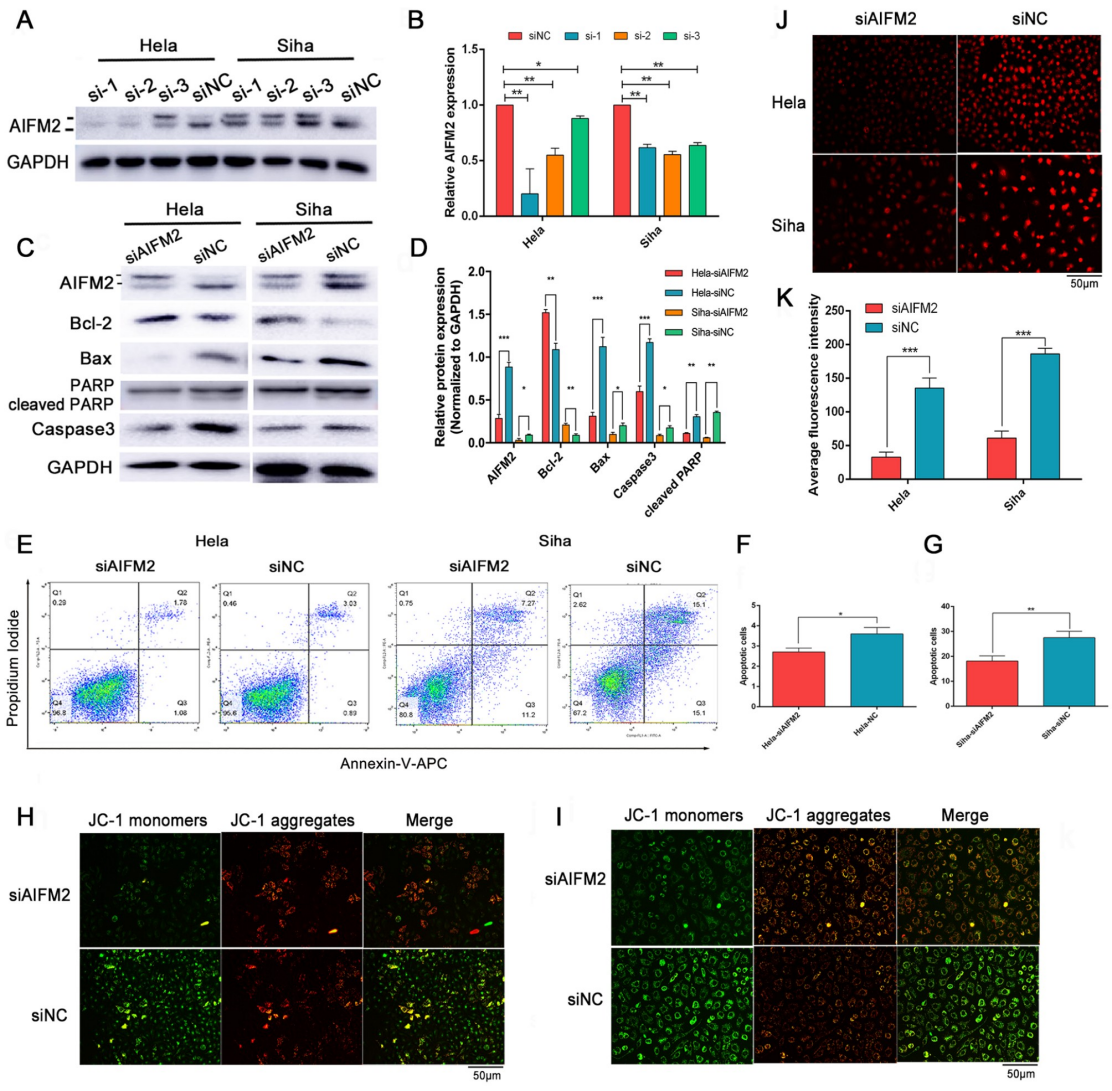
Figure 2 *AIFM2* knockdown represses mitochondrial-dependent apoptosis of HeLa and SiHa cells To analyse the function of AIFM2 in cell apoptosis, we designed three siRNAs to inhibit AIFM2 expression. The inhibitory effects of AIFM2 in HeLa and SiHa cells were validated by western blot analysis (A) and qRT-PCR (B). As si-1 and si-2 have the most prominent effects on HeLa and SiHa cells, we chose these two siRNAs for the subsequent experiments. After inhibiting AIFM2 expression, apoptosis-related proteins were detected and analysed by western blot analysis (C,D), and apoptotic cells were measured and quantified by flow cytometry (E–G). (H,I) The mitochondrial membrane potential of AIFM2-silenced cervical cancer cells and control cells was detected by JC-1 assay. The loss of mitochondrial membrane potential was represented by the fluorescence change from red to green. Scale bar: 50 μm. (J) The intracellular Ca 2+ concentration was detected using the Rhod-2 AM probe. The fluorescence intensity is proportional to the intracellular Ca 2+ concentration. Scale bar: 50 μm. (K) A histogram representing the statistical analysis of the intracellular Ca 2+ concentration. Data are presented as the mean±SD of three replicates. * P<0.05, ** P<0.01, *** P<0.001.

Some articles have proven the crucial role of *AIFM2* in apoptosis, but few studies have focused on its function in cancer cells, including cervical cancer cells. In the above section, we found that *AIFM2* could be upregulated in *IMPA2*-silenced HeLa and SiHa cells. To further explore whether *AIFM2* affects apoptosis in cervical cancer cells, we designed three siRNAs to knockdown *AIFM2* gene expression. As shown in Figure 2A,B, *AIFM2* was significantly downregulated at both the mRNA and protein levels ( *P*<0.01). Among the three siRNAs, si-1 had the most significant inhibitory effect on HeLa cells, and si-2 suppressed most SiHa cells. Based on this, we chose si-1 and si-2 for our subsequent experiments. As shown in Figure 2C,D, *AIFM2*-knockdown cells had lower expression of Bax and Caspase3, consistent with an increase in Bcl-2 expression. Meanwhile, the percentage of apoptotic cancer cells transfected with *AIFM2* siRNA was also decreased compared with that in the control group ( Figure 2E‒G).

On the other hand, in *AIFM2*-knockdown HeLa and SiHa cells, the intensity of green fluorescence was weaker, and the red fluorescence was stronger after JC-1 dye staining, while opposite results were obtained in the control cells. These results indicated a redistribution of the mitochondrial membrane potential ( Figure 2H,I). In addition, *AIFM2-*knockdown cells also had a lower concentration of mitochondrial Ca ^2+^ ( *P*<0.001). These results demonstrated that *AIFM2* could regulate the apoptosis of cervical cancer cells in a mitochondria-dependent manner.

### *AIFM2* inhibition has little effect on cervical cancer progression and survival

To explore the function of AIFM2 in tumors, *AIFM2* expression was first queried in the GCBI database ( http://college.gcbi.com.cn/) ( Supplementary Figure S2A). Then, the analysis of a public CESC (Cervical squamous cell carcinoma) dataset from The Cancer Genome Atlas (TCGA) ( https://tcga-data.nci.nih.gov/) showed that there was no significant difference in *AIFM2* expression between cancer tissues and normal tissues ( Supplementary Figure S2B). The survival rates of patients with high *AIFM2* expression and patients with low *AIFM2* expression were also not obviously different ( *P*=0.55; Supplementary Figure S2C). To confirm the results from the databases, we further detected *AIFM2* expression in 4 pairs of cervical cancer tissues and the corresponding para-carcinoma tissues. No significant difference was found in *AIFM2* expression between cancer tissues and the corresponding para-carcinoma tissues ( Supplementary Figure S2D). Similar to the tissue results, silencing of *AIFM2* in SiHa and HeLa cells had little effect on cervical cancer cell growth ( Supplementary Figure S2E,F). Additionally, *AIFM2* expression in cervical cancer tissues and normal tissues showed no significant difference, as detected by IHC staining ( Supplementary Figure S2G). In summary, *AIFM2* expression may have little effect on cancer progression and survival, which may be explained by the complex functions of AIFM2, including the induction of apoptosis and suppression of ferroptosis.

### *IMPA2* knockdown triggers AIFM2-mediated apoptosis via the p53 signaling pathway in HeLa and SiHa cells

**Figure 3:**
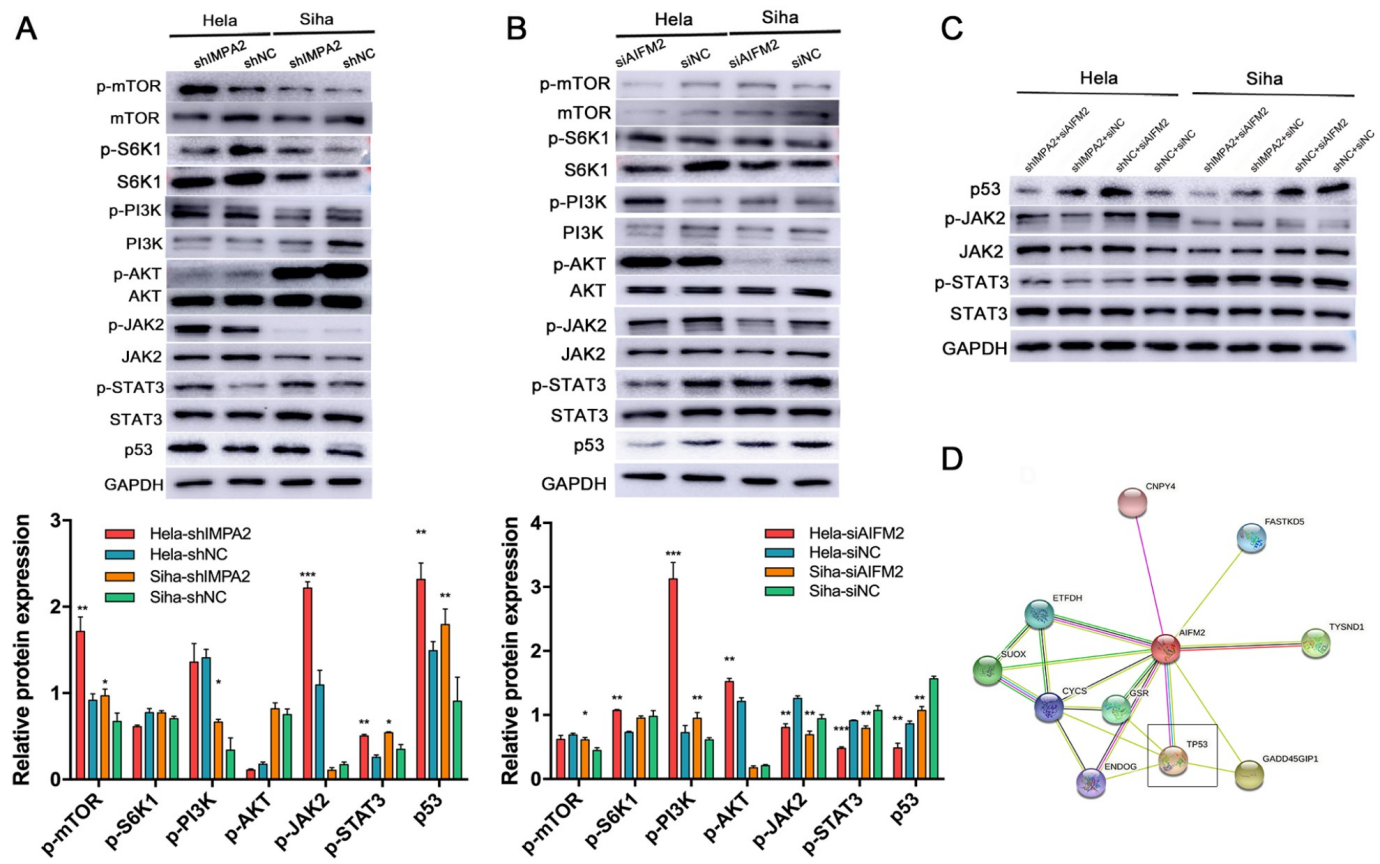
Figure 3 *IMPA2* inhibition regulates AIFM2-mediated cell apoptosis through p53 activation To explore the mechanisms responsible for IMPA2 knockdown-induced cell apoptosis, we screened the classical apoptotic pathway by detecting protein expression after inhibiting IMPA2. Classical pathway proteins were also measured in IMPA2- (A) and AIFM2-knockdown (B) cells and control cells. The ratios of phosphorylated and total protein expression were calculated. Only p53 and JAK2/STAT3 phosphorylation showed significant differences in shIMPA2 and siAIFM2 HeLa and SiHa cells. Therefore, shIMPA2 and siAIFM2 were further cotransfected into cervical cancer cells, and the protein expression levels of p53 and JAK2/STAT3 were detected. Only p53 expression was successfully rescued (C). (D) Database analysis of the relationship between AIFM2 and p53 is shown using the STRING database. Data are presented as the mean±SD of three replicates. * P<0.05, ** P<0.01, *** P<0.001.

To further understand the mechanisms involved in IMPA2-AIFM2-regulated cell apoptosis, we identified some classical pathways of apoptosis regulation in cancer cells. PI3K/AKT/mTOR, JAK/STAT3, and p53 proteins were detected by western blot analysis in *IMPA2*-silenced cells and control cells ( Figure 3A). The results showed that mTOR protein was activated, but PI3K and AKT were only activated in HeLa or SiHa cells, suggesting that PI3K/AKT/mTOR may be partially involved in IMPA2-regulated cell apoptosis. In addition, *IMPA2* silencing significantly increased JAK/STAT3 phosphorylation and p53 expression in SiHa and HeLa cells, indicating that *IMPA2* may affect apoptosis via the JAK/STAT3 pathway or p53 pathway. Additionally, proteins in apoptotic pathways were detected in the *AIFM2*-knockdown cells and control cells. As shown in Figure 3B, JAK/STAT3 phosphorylation was repressed, and p53 expression was also decreased after *AIFM2* gene inhibition. Furthermore, *IMPA2* silencing-induced p53 activation was suppressed by inhibition of *AIFM2*. At the same time, the phosphorylation of JAK/STAT3 was not rescued by siAIFM2 ( Figure 3C), suggesting that p53 may be a key molecule for IMPA2-AIFM2-regulated tumor apoptosis. In addition, the relationship between AIFM2 and p53 was also verified from the STRING database ( Figure 3D).

### PFT-α treatment successfully rescues p53-induced cell apoptosis in *IMPA2*-silenced HeLa and SiHa cells

**Figure 4:**
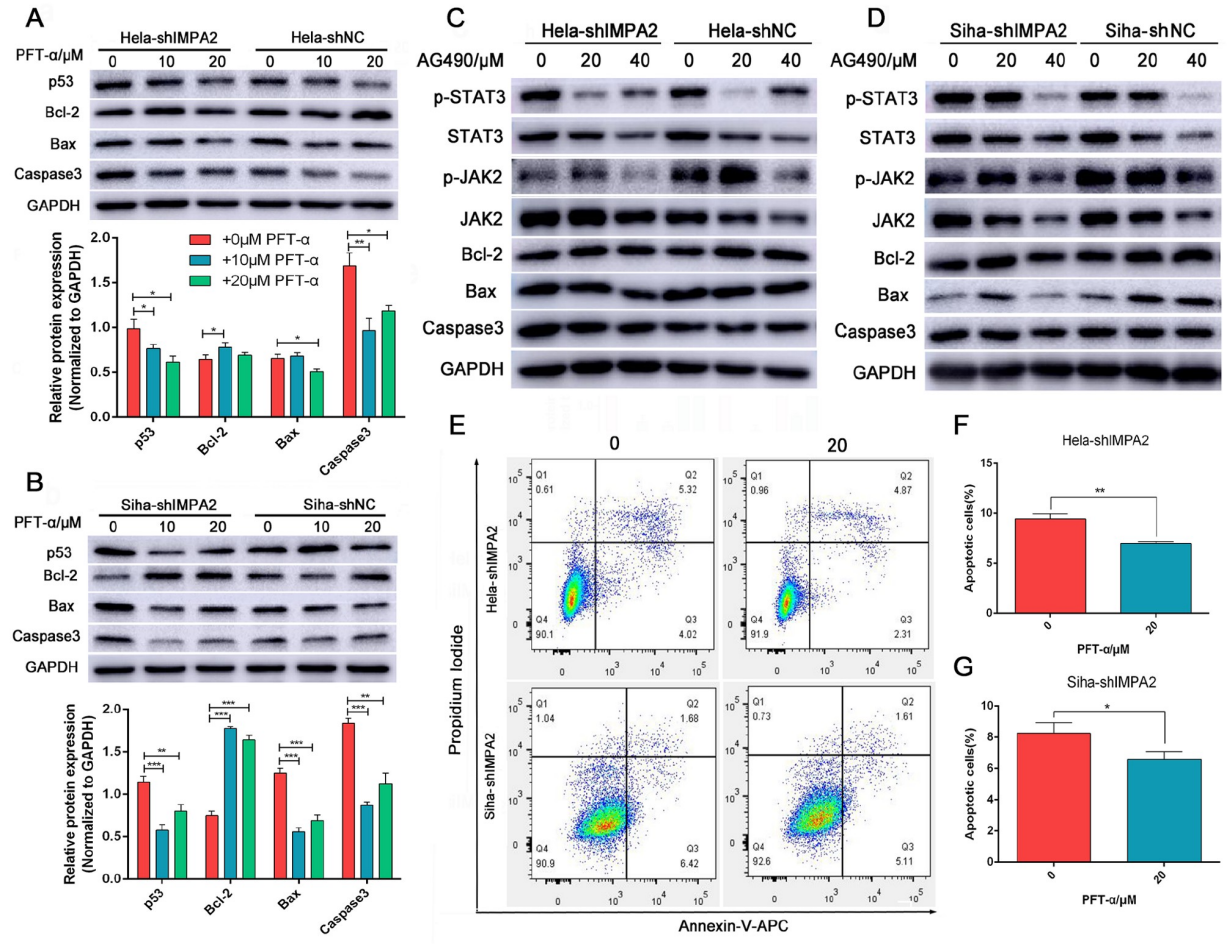
Figure 4 PFT-α treatment rescues *IMPA2* knockdown-induced apoptosis in HeLa and SiHa cells After treatment with different concentrations of p53 inhibitor (PFT-α), apoptosis-related proteins were measured in IMAP2-silenced HeLa (A) and SiHa (B) cells to validate whether PFT-α could reverse IMPA2 knockdown-induced cell apoptosis. Apoptosis-related proteins were also detected in IMPA2-silenced and control HeLa (C) and SiHa (D) cells after treatment with AG490, an inhibitor of JAK2/STAT3. (E–G) Apoptotic cells were also analysed and quantified by Annexin-V-APC staining after 0 and 20 μM PFT-α treatment in IMPA2-silenced HeLa and SiHa cells. Data are presented as the mean±SD of three replicates. * P<0.05, ** P<0.01, *** P<0.001.

To further identify the roles of p53 and JAK/STAT3 in cervical cancer apoptosis induced by IMPA2 and AIFM2, we examined the effects of PFT-α and AG490 on apoptosis proteins. As shown in Figure 4A,B, PFT-α treatment caused *IMPA2*-silenced HeLa and SiHa cells to express lower levels of Bax and Caspase3 and higher levels of Bcl-2, when compared to those in the untreated cells ( *P*<0.05). Similarly, apoptotic cells were significantly decreased in *IMPA2*-knockdown cells after treatment with 20 μM PFT-α in HeLa and SiHa cells, as revealed by Annexin-V-APC/PI staining ( Figure 4E‒G). After AG490 treatment, there were no significant changes in apoptosis proteins compared with the control group ( Figure 4C,D). Overall, *IMPA2* silencing, with activation of AIFM2, promoted apoptosis of cervical cancer cells via the p53 signaling pathway.

### IMPA2 enhances paclitaxel resistance by inhibiting apoptosis in cervical cancer cells

**Figure 5:**
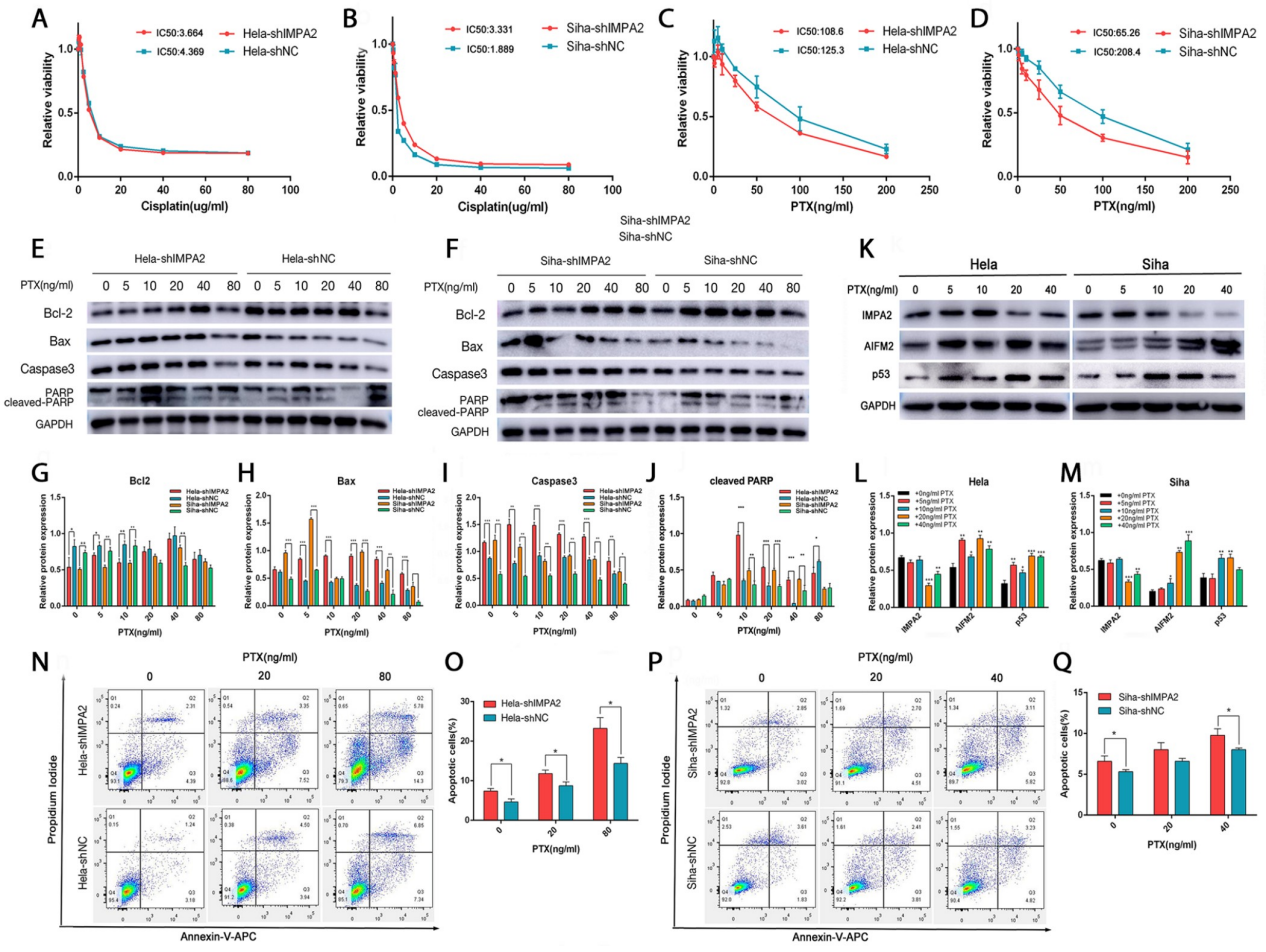
Figure 5 IMPA2 enhances paclitaxel resistance by inhibiting apoptosis in cervical cancer cells CCK-8 assay was used to detect cell activity and calculate the IC 50 values after 24 h of treatment of IMPA2-knockdown HeLa (A) and SiHa (B) cells with different concentrations of cisplatin (C,D). IMPA2-knockdown HeLa (E) and SiHa (F) cells were treated with different concentrations of paclitaxel, and western blot analysis was used to detect the expressions of apoptosis-related proteins. (G‒J) Quantitative analysis of the differences in the expression of Bcl-2, Bax, Caspase3 and cleaved PARP in E and F. (K) After the same treatment in G‒J, the expression levels of IMPA2, AIFM2, and p53 were detected by western blot analysis. (L‒M) Quantitative analysis of the expression differences of each protein in K. After treating HeLa cells (N) with 0, 20, and 80 ng/mL paclitaxel and SiHa cells (P) with 0, 20, and 40 ng/mL paclitaxel, the apoptotic cells were analysed by flow cytometry. (O,Q) The quantification of the proportion of cells undergoing early apoptosis and late apoptosis in N and P. Data are presented as the mean±SD of three replicates. * P<0.05, ** P<0.01, *** P<0.001.

Cellular drug resistance is now a challenge in the clinical treatment of cervical cancer. It has been shown that inhibition of apoptosis is one of the leading causes of tumor chemotherapy resistance. We selected the clinical first-line drugs cisplatin and paclitaxel as the study subjects. We found that knockdown of *IMPA2* had no significant effect on the tumor cell killing effect of cisplatin after IMPA2 expression was inhibited ( Figure 5A,B). However, there was a significant synergistic effect on paclitaxel. After knockdown of *IMPA2*, the IC _50_ values of paclitaxel were decreased in both HeLa and SiHa cells ( Figure 5C,D), indicating that inhibition of IMPA2 expression could enhance the sensitivity of tumor cells to paclitaxel. Then, different concentrations of paclitaxel were used to treat *IMPA2*-knockdown cells and negative control cells, and significant differences in apoptosis-related proteins were found in HeLa cells ( Figure 5E) and SiHa cells ( Figure 5F). Treatment with paclitaxel caused a decrease in anti-apoptotic proteins ( Figure 5G) and an increase in pro-apoptotic proteins ( Figure 5H‒J) compared with the untreated group. Compared with the control cells, the apoptosis of cells was significantly enhanced in the shIMPA2 group after treatment with paclitaxel, indicating that knockdown of *IMPA2* could synergize with the proapoptotic effect of paclitaxel and increase the sensitivity of cervical cancer cells to paclitaxel. Meanwhile, 20 and 40 ng/mL of paclitaxel were also found to significantly inhibit the expression of IMPA2 and upregulate the expressions of AIFM2 and p53 ( Figure 5K‒M), suggesting that paclitaxel may exert its tumor-killing effect by regulating the expression of IMPA2/AIFM2/p53 and that IMPA2 may be a new target for the action of paclitaxel. To further demonstrate that IMPA2 can generate drug resistance by affecting paclitaxel-mediated apoptosis, we selected cervical cancer cells with stable knockdown of *IMPA2* and control cells. We treated them with appropriate concentrations of paclitaxel based on the western blot analysis results. We found that apoptotic cells were increased in cervical cancer cells with *IMPA2* knockdown after treatment with paclitaxel compared with that in control cells ( Figure 5N‒Q). The paclitaxel-induced apoptosis was concentration-dependent in HeLa cells ( Figure 5N,O). This indicates that inhibition of IMPA2 expression can synergize with paclitaxel-induced apoptosis and thus promote the sensitivity of cervical cancer cells to paclitaxel.

## Discussion

Due to an increase in cervical screening and the popularization of the HPV vaccine, deaths from cervical cancer have decreased in recent years. However, the prognosis of cervical cancer remains poor, especially in developing countries [14]. Resistance to chemotherapy drugs contributes to treatment failure or tumor recurrence, and molecular targeted therapy displays a remarkable curative effect. Thus, developing new molecules for cervical cancer screening and treatment is urgently needed. In this study, *IMPA2*, a novel gene we previously found from transcriptomics analysis [11], was proved to be a potential tumor-promoting gene. We further confirmed that *IMPA2* might affect cancer cell death by inducing cell apoptosis. AIFM2, a NAD(P)H-dependent oxidoreductase involved in the cellular oxidative stress response [15], has also been engaged in *IMPA2* knockdown-mediated cell apoptosis by activating p53. This evidence suggests that *IMPA2* is a potential target for cervical cancer therapy.

*IMPA2* irregularity has always been proven to contribute to the pathophysiology of bipolar disorder [ 6, 16, 17] . Few studies have focused on its other functions. In this study, we found that *IMPA2* could affect the survival of cervical cancer cells by disturbing the apoptotic process. It was reported that altered *IMPA2* gene expression is related to calcium homeostasis in bipolar disorder [9], providing indirect evidence of a relationship between *IMPA2* and cell apoptosis.

Although we have confirmed the proapoptotic function of *AIFM2* in cervical cancer, conflicting data exist on the proapoptotic function of the protein in different diseases [ 18– 20] . By comparing the viability of *AIFM2*-silenced cells and control cells, we found that there was no significant difference ( Supplementary Figure S2), indicating that apart from apoptosis, other processes regulated by *AIFM2* may also be involved in cervical cancer cell death. It has been reported that FSP1/AIFM2 is a glutathione-independent ferroptosis suppressor, which acts parallel to glutathione peroxidase 4 (GPX4) to inhibit ferroptosis [ 21, 22] . Ferroptosis is a newly discovered form of regulated cell death, the nexus between metabolism and human health [23]. As *AIFM2* encodes FSP1, which blocks ferroptosis and induces mitochondrial-dependent cell apoptosis, we did not find apparent changes in cell viability after inhibiting *AIFM2* expression. Moreover, to explore whether ferroptosis is involved in *IMPA2*-regulated cell death, we detected GPX4 expression. The results showed that there is no significant difference between *IMPA2*-knockdown cells and control cells ( Supplementary Figure S1), suggesting that IMPA2-induced cancer progression may depend on ferroptosis.

Cisplatin and paclitaxel are the first-line drugs for clinical cervical cancer treatment. With the emergence of clinical drug resistance, the effectiveness of chemotherapeutic drugs decreases. Therefore, exploring the mechanism of drug resistance of chemotherapeutic drugs and enhancing the sensitivity of drugs to cancer cells have become hot spots and focuses of continuous research to improve the survival rate of patients.

*IMPA2*, as a potential oncogene, was well established in this study to regulate the onset of apoptosis in cervical cancer cells. It has also been found that inhibition of *IMPA2* expression can synergize with the tumor-killing effect of paclitaxel. Nevertheless, it had no significant impact on the efficacy of cisplatin ( Figure 5A–D), which is related to the different mechanisms by which cisplatin and paclitaxel exert their antitumour effects, *i.e.*, cisplatin has a nonspecific effect on the cell cycle, while paclitaxel has a specific effect on the cell cycle [ 24, 25] . Many studies have shown that paclitaxel can cause cancer cell death by regulating apoptosis. In addition to causing mitotic cell cycle arrest, paclitaxel can also induce and activate the transcription of various apoptosis-related genes, thereby inducing apoptosis. Our results ( Figure 5) also suggest that paclitaxel can kill tumor cells by regulating the expression of IMPA2/AIFM2, and this mechanism provides a new target for the major role of paclitaxel as an anti-cervical cancer drug in cancer chemotherapy.

In addition, the potent efficacy of p53 in tumor suppression and the high frequency of p53 variants in cancer have stimulated the development of many cancer therapies targeting the p53 signaling network. For example, the success of retinoic acid versus arsenic therapy for acute promyelocytic leukemia (APL) depends on p53-mediated cellular senescence. Although we have revealed many aspects of the p53 signaling network, there is no clear understanding of how p53 performs its multiple functions under certain circumstances, and therefore, it still needs to be studied in detail to improve our understanding of gene regulation mechanisms and nature′s means of defense against cancer. In this study, we found that in addition to the change in IMPA2/AIFM2 expression in paclitaxel-treated cells, the expression of p53 also increased with increasing drug concentration, suggesting that p53 is also involved in regulating paclitaxel-induced apoptosis in cervical cancer cells, i.e., IMPA2/AIFM2/p53 may be a new molecular mechanism for the development of paclitaxel resistance and a potential new target for paclitaxel sensitization therapy.

In conclusion, we identified that *IMPA2* knockdown promotes cervical cancer cell apoptosis, and the mechanistic investigation revealed that *IMPA2* knockdown triggers *AIFM2* expression and activates p53, thereby inducing cancer cell apoptosis. Taken together, our study provides a potential target for cervical cancer therapy.

## Supporting information

378FigS1-S2

## References

[1] Li Z, Wu H, Yi X, Tian F, Zhang X, Zhou H, Liu B (2020). Area-specific economic status should be regarded as a vital factor affecting the occurrence, development and outcome of cervical cancer. Sci Rep.

[2] Sung H, Ferlay J, Siegel RL, Laversanne M, Soerjomataram I, Jemal A, Bray F (2021). Global cancer statistics 2020: GLOBOCAN estimates of incidence and mortality worldwide for 36 cancers in 185 countries. CA Cancer J Clin.

[3] Gadducci A, Cosio S. Pharmacological treatment of patients with metastatic, recurrent or persistent cervical cancer not amenable by surgery or radiotherapy: state of art and perspectives of clinical research.
Cancers (Basel) 2020, 12: 2678. https://doi.org/10.3390/cancers12092678.

[4] Kalliala I, Athanasiou A, Veroniki AA, Salanti G, Efthimiou O, Raftis N, Bowden S (2020). Incidence and mortality from cervical cancer and other malignancies after treatment of cervical intraepithelial neoplasia: a systematic review and meta-analysis of the literature. Ann Oncol.

[5] Derakhshan A, Chen Z, Van Waes C (2017). Therapeutic small molecules target inhibitor of apoptosis proteins in cancers with deregulation of extrinsic and intrinsic cell death pathways. Clin Cancer Res.

[6] Ohnishi T, Yamada K, Ohba H, Iwayama Y, Toyota T, Hattori E, Inada T (2007). A promoter haplotype of the inositol monophosphatase 2 gene (IMPA2) at 18p11.2 confers a possible risk for bipolar disorder by enhancing transcription. Neuropsychopharmacology.

[7] Cryns K, Shamir A, Shapiro J, Daneels G, Goris I, Van Craenendonck H, Straetemans R (2007). Lack of lithium-like behavioral and molecular effects in IMPA2 knockout mice. Neuropsychopharmacology.

[8] Sjøholt G, Ebstein RP, Lie RT, Berle JØ, Mallet J, Deleuze JF, Levinson DF (2004). Examination of IMPA1 and IMPA2 genes in manic-depressive patients: association between IMPA2 promoter polymorphisms and bipolar disorder. Mol Psychiatry.

[9] Yoon IS, Li PP, Siu KP, Kennedy JL, Cooke RG, Parikh SV, Warsh JJ (2001). Altered IMPA2 gene expression and calcium homeostasis in bipolar disorder. Mol Psychiatry.

[10] Lin YF, Chou JL, Chang JS, Chiu IJ, Chiu HW, Lin YF (2019). Dysregulation of the miR-25-IMPA2 axis promotes metastatic progression in clear cell renal cell carcinoma. EBioMedicine.

[11] Zhang K, Liu L, Wang M, Yang M, Li X, Xia X, Tian J (2020). A novel function of IMPA2, plays a tumor-promoting role in cervical cancer. Cell Death Dis.

[12] Lu J, Chen J, Xu N, Wu J, Kang Y, Shen T, Kong H (2016). Activation of AIFM2 enhances apoptosis of human lung cancer cells undergoing toxicological stress. Toxicol Lett.

[13] Tan JH, Cao RC, Zhou L, Zhou ZT, Chen HJ, Xu J, Chen XM (2020). ATF6 aggravates acinar cell apoptosis and injury by regulating p53/AIFM2 transcription in Severe Acute Pancreatitis. Theranostics.

[14] Campos NG, Tsu V, Jeronimo J, Mvundura M, Kim JJ (2017). Evidence-based policy choices for efficient and equitable cervical cancer screening programs in low-resource settings. Cancer Med.

[15] Nguyen HP, Yi D, Lin F, Viscarra JA, Tabuchi C, Ngo K, Shin G (2020). Aifm2, a NADH oxidase, supports robust glycolysis and is required for cold- and diet-induced thermogenesis. Mol Cell.

[16] Bloch PJ, Weller AE, Doyle GA, Ferraro TN, Berrettini WH, Hodge R, Lohoff FW (2010). Association analysis between polymorphisms in the myo-inositol monophosphatase 2 (IMPA2) gene and bipolar disorder. Prog Neuropsychopharmacol Biol Psychiatry.

[17] Jiménez E, Arias B, Mitjans M, Goikolea JM, Roda E, Sáiz PA, García-Portilla MP (2013). Genetic variability at IMPA2, INPP1 and GSK3β increases the risk of suicidal behavior in bipolar patients. Eur Neuropsychopharmacol.

[18] Cho J, Teshigawara R, Kameda M, Yamaguchi S, Tada T (2019). Nucleus‐localized adiponectin is survival gatekeeper through miR‐214‐mediated
*AIFM2* regulation. Genes Cells.

[19] Fan FY, Deng R, Yi H, Sun HP, Zeng Y, He GC, Su Y (2017). The inhibitory effect of MEG3/miR-214/AIFM2 axis on the growth of T-cell lymphoblastic lymphoma. Int J Oncol.

[20] Miriyala S, Thippakorn C, Chaiswing L, Xu Y, Noel T, Tovmasyan A, Batinic-Haberle I (2016). Novel role of 4-hydroxy-2-nonenal in AIFm2-mediated mitochondrial stress signaling. Free Radical Biol Med.

[21] Bersuker K, Hendricks JM, Li Z, Magtanong L, Ford B, Tang PH, Roberts MA (2019). The CoQ oxidoreductase FSP1 acts parallel to GPX4 to inhibit ferroptosis. Nature.

[22] Doll S, Freitas FP, Shah R, Aldrovandi M, da Silva MC, Ingold I, Goya Grocin A (2019). FSP1 is a glutathione-independent ferroptosis suppressor. Nature.

[23] Liang C, Zhang X, Yang M, Dong X. Recent progress in ferroptosis inducers for cancer therapy.
Adv Mater 2019, 31: e1904197. https://doi.org/10.1002/adma.201904197.

[24] Black DJ, Livingston RB. Antineoplastic drugs in 1990. A review (Part I).
Drugs 1990, 39: 489–501. https://doi.org/10.2165/00003495-199039040-00002.

[25] Horwitz SB. Taxol (paclitaxel): mechanisms of action.
Ann Oncol1994, 5 Suppl 6: S3–S6. https://pubmed.ncbi.nlm.nih.gov/7865431/.

