# Supplementary material for: IMPA2 blocks cervical cancer cell apoptosis and induces paclitaxel resistance through p53-mediated AIFM2 regulation: IMPA2 blocks cervical cancer cell apoptosis
Source: Acta Biochim Biophys Sin (Shanghai). 2023 Apr 28;55(4):623–32. doi: 10.3724/abbs.2023069 (PMC10195139; doi:10.3724/abbs.2023069)
Supplement: 378FigS1-S2 [file 378FigS1-S2.pdf]

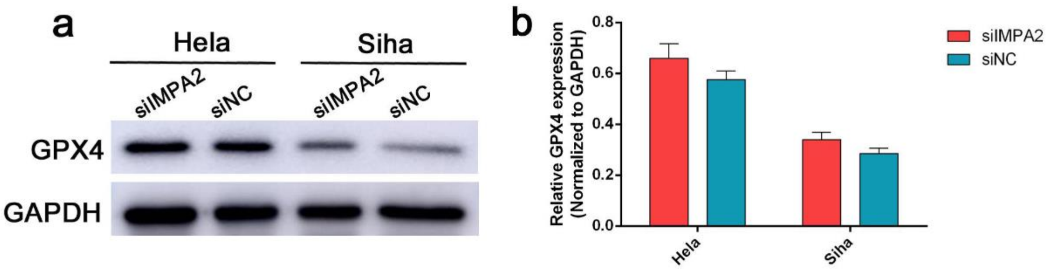

Supplementary Figure S1. The expression of GPX4, a ferroptosis-related protein, was detected by western blotting in *IMPA2*-silenced and control HeLa and Siha cells

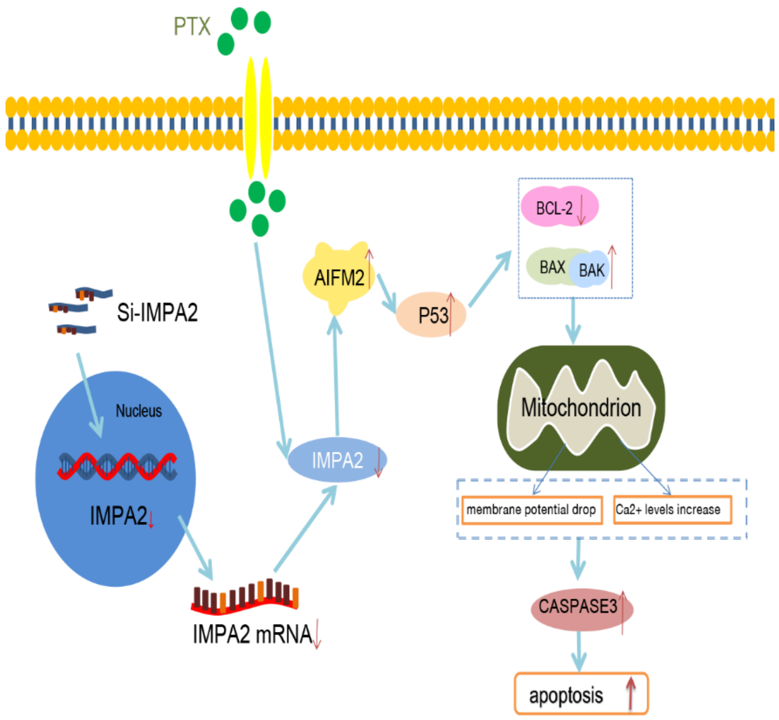

Supplementary Figure S2. AIFM2 expression had little effect on cervical cancer progression and survival (A) AIFM2 expression was demonstrated in different tumor tissues from the GCBI database (http://college.gcbi.com.cn/). (B) Expression levels of AIFM2 in cervical cancer tissues and normal tissues were analysed based on the CESC data set (https://tcga-data.nci.nih.gov/). (C) The effect of AIFM2 expression level on CESC patient survival was analysed (https://tcga-data.nci.nih.gov/). (D) mRNA expression levels of AIFM2 were detected in 4 pairs of cervical cancer tissues and the corresponding para-carcinoma tissues. Cell viability of HeLa (E) and Siha cells (F) transfected with siAIFM2 or siNC was measured by CCK-8 assay. (G) AIFM2 expression was measured and quantified in cervical tissues and normal tissues by IHC staining. Scale bar, 50 μm or 100 μm. Data are represented as the mean ± SD of 3 replicates.
